# Spatiotopic and saccade-specific transsaccadic memory for object detail

**DOI:** 10.1167/jov.20.7.2

**Published:** 2020-07-06

**Authors:** Lukasz Grzeczkowski, Jonathan van Leeuwen, Artem V. Belopolsky, Heiner Deubel

**Affiliations:** Allgemeine und Experimentelle Psychologie, Department Psychologie, Ludwig-Maximilians-Universität München, Germany; Department of Experimental and Applied Psychology, Vrije Universiteit Amsterdam, The Netherlands; Department of Experimental and Applied Psychology, Vrije Universiteit Amsterdam, The Netherlands; Allgemeine und Experimentelle Psychologie, Department Psychologie, Ludwig-Maximilians-Universität München, Germany

**Keywords:** eye movements, transsaccadic integration, blanking effect, change detection, remapping

## Abstract

The content and nature of transsaccadic memory are still a matter of debate. Brief postsaccadic target blanking was demonstrated to recover transsaccadic memory and defeat saccadic suppression of displacement. We examined whether blanking would also support transsaccadic transfer of detailed form information. Observers saccaded to a peripheral, checkerboard-like stimulus and reported whether an intrasaccadic change had occurred in its upper or lower half. On half of the trials, the stimulus was blanked for 200 ms with saccade onset. In a fixation condition, observers kept fixation but the stimulus was displaced from periphery to fixation, mimicking the retinal events of the saccade condition. Results show that stimulus blanking improves transsaccadic change detection, with performance being far superior to the retinally equivalent fixation condition. Our findings argue in favor of a remapped memory trace that can be accessed only in the blanking condition, when not being overwritten by the salient postsaccadic stimulus.

## Introduction

The photoreceptor mosaic of the human retina is highly inhomogeneous, with a much higher density of receptors in the fovea than in the periphery (Curcio, Sloan, Kalina, & Hendrickson, 1990; Østerberg, 1935). This results in high-resolution vision in the very center of the visual field and low-resolution vision in its periphery and is reflected in a decrease of visual performance as a function of retinal eccentricity (Anstis, 1974; Wertheim, 1894). To account for this inhomogeneity, humans make frequent saccadic eye movements (or saccades) to sample the world with the high-resolution fovea. However, before moving the eyes to an object of interest, the visual system must first select that object based on relatively low-resolution information available in the periphery. Thus, the visual system receives at least two different retinal projections of the same object of interest across an eye movement (Herwig, 2015): a peripheral, low-resolution image before the saccade upon which the selection is made and a foveal, high-resolution image after the saccade. To date, it is still unknown how much of the presaccadic image information is transferred across the saccade, what is its nature, and to which extent it is integrated with the postsaccadic image information. Similarly, neural mechanisms underlying the transfer and the integration of presaccadic information are yet largely unknown.

Many previous studies proposed that the transferred information is abstract and relies on working memory (WM), since only relational and structural aspects of the stimulus were found to transfer across saccades (Carlson-Radvansky & Irwin, 1995; McConkie & Zola, 1979; Rayner, McConkie, & Zola, 1980). Moreover, detailed presaccadic information such as stimulus features and location were reported not to transfer across saccades (Bridgeman & Mayer, 1983; Bridgeman & Stark, 1979; Irwin, Brown, & Sun, 1988; Irwin, Yantis, & Jonides, 1983; Irwin, Zacks, & Brown, 1990; Mack, 1970; O'Regan & Lévy-Schoen, 1983; Rayner & Pollatsek, 1983). More recent studies, however, have demonstrated that location (Prime, Niemeier, & Crawford, 2006) and low-level features such as orientation (Fornaciai, Binda, & Cicchini, 2018; Ganmor, Landy, & Simoncelli, 2015; Grzeczkowski, Deubel, & Szinte, 2019; Prime et al., 2006; Stewart & Schütz, 2018a; C. Wolf & Schütz, 2015), spatial frequency (Weiß, Schneider, & Herwig, 2015), and color (Oostwoud Wijdenes, Marshall, & Bays, 2015; Schut, Van der Stoep, Fabius, & Van der Stigchel, 2018; Wittenberg, Bremmer, & Wachtler, 2008) do transfer across saccades and can be integrated with the postsaccadic features.

Recent studies also provided new insights into the nature of the transferred information. For example, Germeys, De Graef, Van Eccelpoel, and Verfaillie (2010) showed that besides relying on WM, transsaccadic memory also relies on another form of memory, which was termed the *visual analog* (De Graef & Verfaillie, 2002). This type of memory is different from WM in that it is maskable and fast decaying. Along similar lines, Zerr et al. (2017) found evidence for spatial remapping of items in a preattentive, high-capacity storage across saccades that precedes WM. In agreement with these results, Edwards, Van Rullen, and Cavanagh (2018) were able to decode signals corresponding to the presaccadic image removed from the screen at saccade onset. Accordingly, such transferred signals were found to fuse perceptually with the postsaccadic low-contrast signals (Paeye, Collins, & Cavanagh, 2017). In summary, growing evidence suggests that the information transferred across the saccade is not solely abstract and restricted to WM but also relies on a high-capacity, maskable, and relatively detailed sensory representation.

Important in the context of the present work, it was also shown that a simple experimental manipulation, the blanking of the saccade target for a brief period (∼ 200 ms) after the saccade, allows one to access precise information about target location and to defeat saccadic suppression of displacement (Deubel, 2004; Deubel, Bridgeman, & Schneider, 1998; Deubel, Schneider, & Bridgeman, 1996, 2002; Gysen, Verfaillie, & De Graef, 2002; Matsumiya, Sato, & Shioiri, 2016; Wexler & Collins, 2014). Therefore, blanking seems to provide a powerful tool for directly studying the content and nature of transsaccadic memory. Recently, we used this blanking paradigm to show that information about a target's orientation is transferred across saccades and can be used for transsaccadic discrimination of orientation changes (Grzeczkowski et al., 2019). To explain our findings, we proposed that a remapped memory trace of the presaccadic retinal image is formed as a result of predictive coding and imagery, which is usually not perceived since it is masked by the salient postsaccadic image (Tas, Moore, & Hollingworth, 2012; Tas, Moore, & Hollingworth, 2014). Due to the introduction of the temporal blank (or of a weak, isoluminant postsaccadic stimulus; Grzeczkowski et al., 2019), this masking is postponed, making a phantom percept of the presaccadic stimulus available.

We here adapted the blanking paradigm introduced by Deubel, Schneider, and Bridgeman (2002) to verify that details of visual form information can indeed be transferred across saccades. We investigated whether this memory trace is specific for eye movements or results from a more general visual mechanism, also present at fixation. Finally, we asked whether the coding of such maskable memory operates in retinotopic or spatiotopic coordinates. To anticipate our results, we found a blanking effect for detailed form, confirming previous results (Deubel et al., 2002). Importantly, we also found that this blanking effect occurs in a spatiotopic frame of reference and only in the presence of a saccade.

## Materials and methods

### Participants

Fifteen naive participants (mean age, 24.93 years; range, 20–31 years; six females) completed the experiment. All participants signed informed consent before the experiment and were compensated with 10€/hour. The study was approved by the Ethics Committee of the Faculty for Psychology and Pedagogics of the Ludwig-Maximilians-Universität München (approval number 13_b_2015) and conducted in accordance with the Declaration of Helsinki.

### General setup

Participants sat in a quiet and dimly illuminated room. Chin and forehead rests were used to minimize head movements. The experiment was controlled by a PC. Gaze position of the dominant eye was recorded using an EyeLink 1000 (SR Research Ltd., Ontario, Canada) with a sampling rate of 1,000 Hz. Stimuli were displayed on a VIEWPixx, LCD monitor (VPixx Technologies, Inc., Saint-Bruno, Canada) at a 1,920 × 1,080–pixel resolution (screen size 515 by 290 mm) and a 120-Hz refresh rate. The monitor was linearized with a Minolta CS-100 luminance meter (Osaka, Japan). Viewing distance was 60 cm. Participants’ responses were recorded via a standard keyboard. The display, response collection, and eyetracking were controlled using MATLAB (MathWorks, Natick, MA) with the Psychophysics Toolbox (Brainard, 1997; Pelli, 1997) and EyeLink toolboxes (Cornelissen, Peters, & Palmer, 2002).

### Stimuli

**Figure 1.**
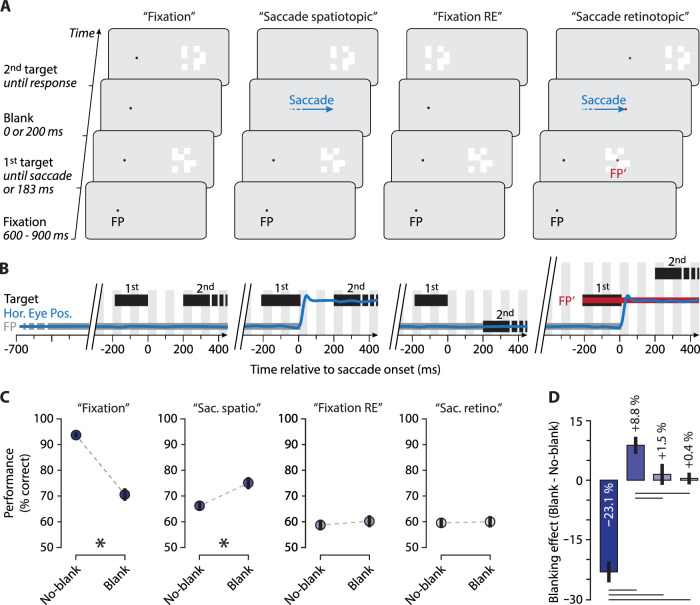
(A) Procedure. After fixating the fixation point (FP), a checkerboard-like stimulus was shown to participants either to the left or right (right only in the figure). In the fixation conditions (first and third columns), the first target was presented for 180 ms, followed by the second target either immediately (no-blank condition) or after a 200-ms blank (only the blank condition is shown in the figure). The second target contained a small change in the checkerboard's form, either in its upper or lower half (upper in the figure). Participants indicated in which half the change occurred. In the saccade conditions (second and fourth columns), the task was the same, except that participants saccaded to the first (presaccadic) target and the target's form change occurred at saccade onset. Similarly, the target was either blanked at the saccade onset or was not blanked. (B) Time course for each condition. The blue line symbolizes the horizontal eye position (Hor. Eye. Pos.) in a typical trial containing a blank. Black rectangles illustrate the timing of the first and second target. The gray and red rectangles illustrate the fixation points (FP and FP′). (C) Mean performance for no-blank and blank conditions. Expectedly, blanking deteriorated performance in the fixation condition (first panel). Significant improvement in performance due to blanking was observed only in the condition where the first and second targets were presented in the same spatiotopic location and in the presence of a saccade (second panel). Asterisks denote significant effects (corrected). (D) Blanking effect as performance difference between blank and no-blank for each condition. Black horizontal lines denote significant effects (corrected). Error bars show ± 1 *SEM*.

Stimuli were checkerboard-like patterns (Phillips, 1974) presented on a gray (21 cd/m^2^) background. The patterns were composed of eight white squares (∼ 80 cd/m^2^, 0.5 degrees of visual angle [dva] each), arranged within a 4 × 4 matrix, two squares per each matrix line. The whole checkerboard-like pattern was 2 × 2 dva. In each trial, the arrangement of the white squares was random but ensuring that both the upper and lower halves of the checkerboard-like pattern contained four white squares each. In each trial, the initially presented checkerboard was replaced, either during the saccade or after a fixed delay (see below), by a second checkerboard that was similar to the first except that either in its upper or lower half, one randomly chosen white square was displaced (Figure 1A). The fixation point was a black (∼0 cd/m^2^) dot with a radius of 0.10 dva. In the saccade-retinotopic condition (see below), another red (∼10 cd/m^2^) fixation point (Figure 1A,B; FP′) of the same size was embedded at the center of the first target and remained at that position throughout the trial. This served to stabilize postsaccadic fixation and helped to avoid secondary saccades.

### Procedure

The experiment comprised four different tasks (fixation, saccade-spatiotopic, fixation–retinally equivalent [fixation-RE], and saccade-retinotopic) that were run in separate blocks in a randomized order per individual. In 50% of the trials, the target was blanked for 200 ms (blank condition), and in the other 50%, no blank was introduced (no-blank condition).

At the beginning of each trial, participants were presented with a fixation point at the center of the screen that they had to fixate for at least 200 ms within a virtual circle of 2 dva of radius. The fixation remained present for another randomly chosen duration between 400 and 700 ms that varied from trial to trial. The initial checkerboard was then presented at a distance of 7 dva, randomly either to the left or to the right from the fixation point. The four different tasks differed as follows:

In the saccade-spatiotopic task (Figure 1A,B, second column), participants saccaded to the target's center. Saccade onset was defined as the first gaze sample position outside of a virtual circle of 2 dva of radius around the fixation. Triggered with saccade onset, the initial checkerboard was removed from the screen and replaced by the second, modified checkerboard, either immediately (no-blank condition) or after a delay of 200 ms (blank condition). The position of the second target was the same as that of the first, presaccadic target.

The saccade-retinotopic task (Figure 1A,B, fourth column) was identical to the saccade-spatiotopic task except that the second target now appeared 7 dva away from the first target location, in the direction of the saccade. This implies that the second target was shown at the same retinal (rather that spatial) location as the first stimulus.

In the fixation task (Figure 1A,B, first column), participants were asked to keep central fixation. The initial checkerboard was presented 7 dva in the periphery, to the left or the right of fixation. After a delay of 183 ms, the first stimulus was replaced by the second, modified checkerboard stimulus, either immediately (no-blank condition) or after a 200-ms delay (blank condition).

Finally, in the fixation-RE task (Figure 1A,B, third column), the second target was shown in the center of the screen, replacing the fixation point. Again, the second target could appear immediately after the offset of the initial target (no-blank condition) or after a 200-ms delay (blank condition). In this task, except from the central dot presented during the blank to ensure fixation, the visual events at the observer's retina were similar to the saccade-spatiotopic condition but without the execution of a saccade.

As explained above, the form of the second stimulus differed as compared to the initial one in that a change occurred in the upper or lower half of the checkerboard-like pattern. Participants reported if the target changed in the upper or lower half by pressing the upper or lower arrow of the keyboard, respectively. In all four tasks, the second target remained on the screen until the response. The next trial started after a delay of 1,000 ms following the button press.

Trials in which participants saccaded outside of a 2-dva radius area centered on the target, executed their saccades earlier than 50 ms or later than 750 ms after target onset, or blinked during the trial were repeated at the end of the same block. On average, 245.87 trials (38.42%) were repeated according to these online rejection criteria per participant.

In order to familiarize the participants with the setup and the task, each participant performed 40 training trials in the saccade-spatiotopic task at the beginning of the experiment. During this familiarization, an auditory feedback signal was provided for a wrong button press. After training, participants performed four blocks, each corresponding to one of the four experimental tasks. The order of these blocks was counterbalanced across participants. Participants performed 160 correct trials in each task considering the online rejection eye recording criteria. In summary, participants performed 20 trials × 2 change types (upper vs. lower half) × 2 locations (left vs. right) × 2 blank conditions (blank vs. no-blank) × 4 task types (i.e., 640 correct trials in total). Participants were allowed to take breaks. The experiment lasted about 1 hr.

### Data preprocessing

Before statistical analysis, the eye movement data were preprocessed offline. Saccades were detected based on their velocity distribution using a moving average over 20 subsequent eye position samples (Engbert & Mergenthaler, 2006). Saccade onset and offset were detected when the velocity exceeded and fell behind the median of the moving average by 3 *SD*s for at least 20 ms. Trials were excluded if the fixation was not maintained within a 2.0-dva radius centered on the fixation target or if the saccade did not land within a 2.0-dva radius centered on the postsaccadic stimulus. On average, 637.00 trials were included per participant (99.53%) based on this offline data preprocessing.

### Data analysis

Discrimination performance in the psychophysical task was determined as the percentage of correct reports (change occurred in upper or lower half). Blanking effect denoted performance as a difference between the blank and no-blank conditions, raised to percentage (i.e., blank – no-blank). The blanking effect was first calculated for each participant, then averaged.

For each statistical comparison, 10,000 bootstrap samples were drawn (with replacement) from the original pair of compared values. Then, the difference of these bootstrapped distributions was calculated. A test statistic value from that difference distribution was calculated (mean/standard error) and compared to a normal distribution to obtain a two-tailed *p* value. Because 10 statistical comparisons were performed in the study, the original *p* value thresholds underwent a false discovery rate correction. After correction, 7 of 10 significant comparisons remained significant. The data are available on the Open Science Framework platform: https://osf.io/whr47.

## Results

We first measured the performance at discriminating changes in the target's form in the fixation task. Unsurprisingly, performance to discriminate between changes in the upper or lower half of the checkerboard-like stimulus was very good in the no-blank condition (Figure 1C, first panel; mean = 93.69% ± 1.77%, 95% CI [90.83%, 96.56%]). However, interrupting the presentation of the target by introducing a 200-ms blank (blank condition) strongly and significantly deteriorated task performance (mean = 70.57% ± 2.44%, 95% CI [67.17%, 73.91%]; *p* < 0.0001, *d* = 3.18). Performance was reduced by 23.12% ± 2.64% as compared to the no-blank condition (Figure 1D, first bar).

Second, we determined the performance when participants made an eye movement to the target while its form changed across the saccade (saccade-spatiotopic task). We found poor performance in the no-blank condition (Figure 1C, second panel; mean = 66.20% ± 1.77%, 95% CI [63.93%, 68.51%]). However, the introduction of a blank at the saccade onset and during and after the saccade strongly improved performance (mean = 75.02% ± 2.36%, 95% CI [72.13%, 77.89%]; *p* = 0.0018, *d* = 1.09). This represents an improvement of 8.82% ± 2.14% (Figure 1D, second bar).

Third, we tested whether for fixation, a similar retinal stimulation as in saccade-spatiotopic task, which means an initial peripheral target and a subsequent foveal target, would result in a similar blanking effect or whether the blanking effect is restricted to saccades. We found that the poor yet above-chance performance in the no-blank condition (Figure 1C, third panel; mean = 58.76% ± 2.07%, 95% CI [57.26%, 62.01%]) was improved by only 1.47% ± 2.65% (Figure 1D, third bar) as compared to the blank condition, which was nonsignificant (mean = 60.23% ± 2.32%, 95% CI [58.62%, 62.45%]; *p* = 0.6363, *d* = 0.17). This indicates that the blanking effect for discriminating transsaccadic form changes is saccade specific.

Fourth, we determined performance in the saccade-retinotopic task in which the target appeared at the same retinotopic location before and after the saccade (i.e., the pre- and the postsaccadic targets were both presented in the retinal periphery). Performance was low in both the no-blank (Figure 1C, fourth panel; mean = 59.56% ± 1.71%, 95% CI [58.26%, 62.01%]) and the blank conditions (mean = 59.99% ± 2.23%, 95% CI [57.62%, 62.45%]), with a nonsignificant difference between the two conditions (*p* = 0.8674, *d* = 0.06) and a blanking effect of 0.43% ± 1.48% (Figure 1D, fourth bar).

Finally, we compared the blanking effects between the four conditions (Figure 1D). The improvement due to blanking in the saccade-spatiotopic condition (8.82% ± 2.14%, 95% CI [6.7%, 11.47%]) was significantly higher than in the fixation-RE (1.47% ± 2.65%, 95% CI [–2.12%, 5.32%]; *p* = 0.0246, *d* = 0.79), saccade-retinotopic (0.43% ± 1.48%, 95% CI [–2.61%, 3.63%]; *p* = 0.0008, *d* = 1.18), and fixation (–23.12% ± 2.64%, 95% CI [–27.18%, –18.88%]; *p* < 0.0001, *d* = 3.43) conditions. The (negative) blanking effect in the fixation condition was significantly different from the saccade-retinotopic (*p* < 0.0001, *d* = 2.84) and the fixation-RE (*p* < 0.0001, *d* = 2.40) conditions. Unsurprisingly, the difference between the blanking effects in the saccade-retinotopic and fixation-RE conditions was nonsignificant (*p* = 0.7246, *d* = 0.13).

Saccade latencies (153.22 ± 6.47 ms and 156.23 ± 5.53 ms), amplitudes (7.14 ± 0.22 dva and 7.22 ± 0.37), and durations (49.67 ± 6.83 ms and 48.59 ± 6.34 ms) were similar for both the saccade-spatiotopic and saccade-retinotopic conditions, respectively (means ± standard deviations). Finally, the durations of the presaccadic stimuli were 172.55 ± 6.71 ms and 175.38 ± 5.46 ms for the spatiotopic and retinotopic conditions, respectively. Hence, these durations were equivalent to the durations of the first target in both fixation conditions, which was set to 183 ms. For each condition, the postsaccadic stimuli durations that lasted until the response collection were 613.15 ± 177.30 ms (fixation), 677.11 ± 205.29 ms (saccade-spatiotopic), 742.39 ± 341.33 ms (fixation-RE), and 832.46 ± 337.31 ms (saccade-retinotopic).

## Discussion

The blanking effect as previously reported by Deubel and colleagues is the improvement of performance for discriminating target displacements across eye movements by interrupting the target's presentation for a short period of time (100–250 ms) at the saccade onset (Balp, Waszak, & Collins, 2018; Deubel, 2004; Deubel et al., 1998, 1996, 2002; Gysen et al., 2002; Matsumiya et al., 2016; Wexler & Collins, 2014). The blanking effect demonstrates that information about target location is not lost due to saccadic suppression (Bridgeman, Hendry, & Stark, 1975; Bridgeman & Stark, 1979) but is transferred across eye movements and available after the saccade for perceptual judgments. As it is for target location, it was shown that blanking allows access also to transsaccadic information about stimulus orientation (Grzeczkowski et al., 2019), spatial frequency (Weiß et al., 2015), and form (De Graef & Verfaillie, 2002; Deubel et al., 2002; Germeys et al., 2010). Here, we expand these previous reports demonstrating a blanking effect for the detailed form of the target, and we show that the blanking effect occurs only in spatiotopic coordinates and in the presence of a saccade.

First, our results demonstrate that target blanking causes a drastic deterioration of performance (∼23%, *d* > 3) when participants kept the eyes on central fixation and the second target was presented in the same location (Figure 1A–C, first column). Such an effect is expected: During fixation and without blanking, the visual system can rely on sensitive visual motion detectors, reliably signaling motion either in the upper or lower half of the checkerboard. With a 200-ms blank, however, such motion signals are no longer available and the task then must involve a comparison of a memory trace with the visual input, which leads to a deterioration of discrimination performance similar to the change blindness phenomenon (O'Regan, Deubel, Clark, & Rensink, 2000; Rensink, O'Regan, & Clark, 1997, 2000; Zuiderbaan, van Leeuwen, & Dumoulin, 2017). This result establishes a baseline showing that the task is very feasible in the periphery.

Second, importantly, however, we show an opposite pattern of results when the same stimuli were presented but participants made a saccade to the target. Here, blanking strongly improved performance by ∼8% (*d* > 1) as compared to the no-blank condition (Figure 1A–C, second column), confirming previous reports (Deubel et al., 2002). Hence, not only the information related to position (Deubel et al., 1996), orientation (Grzeczkowski et al., 2019), and spatial frequency (Weiß et al., 2015) of a target but also the details of its form can be better discriminated with the blanking paradigm. This result, together with the previous findings (Deubel et al., 1998, 1996, 2002; Fornaciai et al., 2018; Ganmor et al., 2015; Germeys et al., 2010; Grzeczkowski et al., 2019; Prime et al., 2006; Stewart & Schütz, 2018a, 2019; Weiß et al., 2015; C. Wolf & Schütz, 2015; Zerr et al., 2017), is in obvious contrast to the view that the information transferred across the saccade is abstract, imprecise, and restricted to WM (Bridgeman & Mayer, 1983; Bridgeman & Stark, 1979; Carlson-Radvansky & Irwin, 1995; Irwin et al., 1988, 1990; Jonides, Irwin, & Yantis, 1983; Mack, 1970; McConkie & Zola, 1979; O'Regan & Lévy-Schoen, 1983; Rayner et al., 1980).

Third, we also determined performance in a saccade-mimicking, fixation condition (Figure 1A–C, third column) where participants were first shown a peripheral and then a foveal target while fixating. Importantly, the retinal stimulation in this task was equivalent to the previous saccade condition where a strong blanking effect occurred. Here, however, performance was equally low in both the blank and no-blank conditions, demonstrating that the blanking effect occurs only in the presence of a saccade. Correspondingly, it was reported that integration of target's orientation found across saccades does not occur under such retinally equivalent conditions (e.g., Ganmor et al., 2015). Together, these results demonstrate that mechanisms underlying the transfer of object details across retinal locations are different at fixation from when a saccade is executed.

Fourth, there was no blanking effect in the task where participants executed a saccade to a target while the latter was further displaced in the direction of the saccade at saccade onset. This suggests that the transfer of the presaccadic information occurs in spatiotopic rather than in retinotopic coordinates. This result is in line with recent studies showing that across saccades, motion information updating (Fabius, Fracasso, & Van Der Stigchel, 2016) and orientation integration (Stewart & Schütz, 2019) occur in spatiotopic rather than retinotopic coordinates.

The present results extend our previous findings, which demonstrated a transfer of visual target features such as orientation across the saccade (Grzeczkowski et al., 2019). Similarly as for the target's orientation, the access to this transsaccadic memory is hampered because it is overwritten by, or integrated with, the postsaccadic stimulus that is usually immediately present at the saccade offset. Blanking, however, postpones the overwriting and/or the integration and creates a short temporal window enabling the access to this transsaccadic memory. Its nature differs from WM, because it is maskable by the postsaccadic stimulus and codes the target's details rather than abstract presaccadic information in a similar way as it was found for the target's orientation (Grzeczkowski et al., 2019). In agreement with our results, the existence of such a maskable, pre-WM transsacadic memory has been proposed before (De Graef & Verfaillie, 2002; Edwards et al., 2018; Germeys et al., 2010; Paeye et al., 2017; Zerr et al., 2017). Interestingly, Zerr et al. (2017) proposed that transsacadic integration might occur at different stages of visual processing and employ distinct integration mechanisms at different levels. Specifically, the authors proposed that transsaccadic integration can be driven by a remapped, preattentive, and maskable memory as well as by WM that is capacity limited, attention demanding, and nonmaskable. In accordance with that proposition, recent studies showed that transsacadic integration can be modulated by attention (Stewart & Schütz, 2018a) and be impaired by additional WM load (Stewart & Schütz, 2018b). Nevertheless, because WM is capacity limited, transsaccadic integration cannot rely solely on WM. In accordance with Zerr et al. (2017), a recent study using electroencephalography (EEG) showed evidence for different transsacadic integration processes occurring at different processing stages (Huber-Huber, Buonocore, Dimigen, Hickey, & Melcher, 2019). The study demonstrated that signals relative to the pre- and postsaccadic face targets interact for the first time after saccade landing (170 ± 80 ms) and, later on, for a second time (320 ± 40 ms). These results suggest the existence of fast, pre-WM integration mechanisms, followed by integration at a WM stage. Additionally, the study found evidence in the EEG signal for transsaccadic memory present immediately after saccade. Early studies suggested that a complete, spatiotopic memory trace of the visual world is maintained across the saccade and is the basis of visual stability (Jonides, Irwin, & Yantis, 1982; W. Wolf, Hauske, & Lupp, 1978, 1980). In these studies, observers often reported seeing a postsaccadic stimulus, even when the latter had been removed from the screen during the saccade (Jonides et al., 1982; W. Wolf et al., 1980). While this initial proposal of the existence of a visible, spatiotopic memory was criticized for methodological reasons such as the involvement of display phosphor persistence (Bridgeman & Mayer, 1983; Irwin et al., 1983, 1990; O'Regan & Lévy-Schoen, 1983; Rayner & Pollatsek, 1983), a new line of evidence, not suffering from such problems, demonstrated the existence of transsaccadic integration (Fornaciai et al., 2018; Ganmor et al., 2015; Germeys et al., 2010; Hübner & Schütz, 2017; Oostwoud Wijdenes et al., 2015; Stewart & Schütz, 2018a; C. Wolf & Schütz, 2015) and a possibility of such a perceptual rather than representational information (Edwards et al., 2018; Grzeczkowski et al., 2019; Paeye et al., 2017).

Recently, we proposed that transsaccadic transfer of visual features may rely on such remapped memory that creates a phantom-like percept (Grzeczkowski et al., 2019) as a result of transsaccadic predictive mechanisms and imagery. Models of predictive coding assume that visual stimuli are first processed in a feedforward manner and then fed back from high-level to low-level neurons to form visual predictions (e.g., Rao & Ballard, 1999). Similar processes occur in mental imagery, where high-level processes generate activations in low-level neurons, giving rise to phantom-like percepts (Pearson, Clifford, & Tong, 2008). Hence, visual imagery can lead to the formation of phantom-like percepts that can bias future perception (Pearson et al., 2008; Sterzer, Frith, & Petrovic, 2010) and even replace the real visual stimulus and lead to visual perceptual learning (Grzeczkowski, Tartaglia, Mast, & Herzog, 2015; Shibata, Watanabe, Sasaki, & Kawato, 2011; Tartaglia, Bamert, Mast, & Herzog, 2009). Accordingly, it was shown that stimulus-driven and imagery-driven representations have overlapping neural topography and induce similar activations in early visual areas that are retinotopically organized and feature specific (Naselaris, Olman, Stansbury, Ugurbil, & Gallant, 2015; Slotnick, Thompson, & Kosslyn, 2005). Besides voluntarily visual imagery, automatic, phantom-like percepts such as filling-in in the “neon color spreading” phenomenon were shown to generate neural activity in V1 corresponding to these phantom-like, filling-in percepts (Sasaki & Watanabe, 2004; for a review, see Pearson & Westbrook, 2015). If low-level visual neurons are top-down activated via transsacadic predictions, these activations are likely to create phantom-like percepts in a similar manner. Accordingly, recent evidence has shown that across saccades, predictable targets are better detected (Huber-Huber et al., 2019; Vetter, Edwards, & Muckli, 2012) and produce a decreased BOLD (Blood-oxygen-level-dependent) activity in the early visual cortices as compared to unpredictable targets (Edwards, Vetter, Mcgruer, Petro, & Muckli, 2017; Fairhall, Schwarzbach, Lingnau, Van Koningsbruggen, & Melcher, 2017).

In the study by Poth, Herwig, and Schneider (2015), participants were presented with monochromatic ellipsoid presaccadic stimuli embedding an irrelevant special character (e.g., “&”) that the participants had to saccade to. During the saccade, the whole target changed position, and the character was replaced by a single letter. Interestingly, results demonstrated that while improving the detection of target displacement, blanking deteriorated the identification of the postsaccadic letter contained in that target. This suggests that in the no-blank condition, the remapped memory trace is overwritten by the postsaccadic target. However, similarly to the present study, blanking allows a better access to the memory trace, which interferes with the report of the incongruent postsaccadic stimulus. Interestingly, in subsequent experiments, the authors showed that a transsacadic polarity change in the no-blank condition had similar deteriorating effects on letter identification as blanking (Poth et al., 2015; Poth & Schneider, 2016). Poth and colleagues interpreted these results in the light of the theory of task-driven visual attention and working memory (Schneider, 2013). The theory postulates that pre- and postsaccadic objects compete for attentional processing resources if the object correspondence is broken, for example, by blanking or polarity change. We think their results can be interpreted differently, however. In our view, these findings strongly support the assumption that the transsaccadic memory is of a phantom-like rather than of a representational (Germeys et al., 2010) or an abstract nature. That is, while a polarity change results in a negative postsaccadic image as compared to the presaccadic one, it contains the same representational content. Hence, the integration at a representational level should be independent of a polarity change. On the contrary, an integration of a phantom-like percept with its own negative should to some extent result in canceling and performance deterioration.

We propose that the visible, spatiotopic persistence (Paeye et al., 2017) and the visual analog (De Graef & Verfaillie, 2002; Germeys et al., 2010) do in fact reflect the existence of such phantom-like percepts that result from predictive, transsaccadic mechanisms. We suggest that presaccadic stimuli generate a feedforward propagation of the signal to higher-level areas and that the signal is fed back to early visual neurons at predicted world-centered locations (for static stimuli). This back-propagation occurs during the saccade and ends ∼50 ms after the saccade offset. Accordingly, 50 ms after saccade offset, the detection advantage of target predictability (Vetter et al., 2012) and the blanking effect for target displacements (Deubel & Schneider, 1996) begin to be effective, and event-related potentials in the parietal posterior cortex reflecting the transsaccadic integration processes can be distinguished (Bellebaum & Daum, 2006). Such transsaccadic predictive mechanisms would offer an important advantage in stimulus processing speed as the postsaccadic stimulus signal needs time to travel from the retina and undergo subsequent processing (Thorpe, Fize, & Marlot, 1996). For example, the earliest visual evoked potential peaks (P1, C1) can usually be observed only about 100 ms after stimulus onset (Luck, 2005). This is in line with studies showing that a presaccadic preview of a saccade target allows faster target identification (Crouzet, 2010; Henderson & Anes, 1994; Huber-Huber et al., 2019; Schotter, Ferreira, & Rayner, 2013) and reading (Rayner, Slattery, Drieghe, & Liversedge, 2011). The predictive activations from the high-level areas would excite low-level neurons, which, similarly to visual imagery, result in a phantom-like percept. This percept and its underlying neural activity ensure the visual continuity of the target despite the interruption of visual input due to saccadic suppression. Once the saccade has ended, the processing of the postsaccadic stimulus is achieved, and it integrates with or overwrites the phantom percept of the presaccadic stimulus. Hence, the phantom percept is usually not visible unless the postsaccadic stimulus presentation is postponed by at least 50 ms as it is the case with blanking. If the postsaccadic stimulus is of low contrast or is isoluminant, the integration relies more on the phantom percept or it does not succeed in overwriting it (Grzeczkowski et al., 2019; Matsumiya et al., 2016). Accordingly, it was proposed that transsaccadic integration occurs in an optimal integration way as a weighted sum of the reliabilities of pre- and postsaccadic stimuli (Ganmor et al., 2015; Oostwoud Wijdenes et al., 2015; C. Wolf & Schütz, 2015). For example, C. Wolf and Schütz (2015) demonstrated that the reliability of the postsaccadic stimulus and its level of integration with the presaccadic stimulus are affected by a postsaccadic target contrast manipulation. Alternatively, it has also been suggested that the transsaccadic integration of target location might be explained with a Bayesian causal inference model (Atsma, Maij, Koppen, Irwin, & Medendorp, 2016). The model evaluates the probability of determining whether the transsacadic memory signal and the postsaccadic visual signal belong to the same or to different objects. If both signals are considered as belonging to the same object, they are integrated. On the contrary, integration does not occur if the signals are considered as belonging to two different objects. Here, we provide an explanation of how one of the model inputs (i.e., the transsaccadic memory) could be created in first place.

In conclusion, by using the blanking paradigm, we showed that detailed form information is transferred across eye movements. Moreover, we demonstrated that the transferred memory trace operates in spatiotopic coordinates and depends on the execution of a saccade. These results are in line with recent studies showing transsaccadic transfer and integration of visual features. They further support the view that this form of transsaccadic memory precedes WM and generates a phantom-like percept, which, when made accessible by blanking, improves the detection of visual changes that occur across the saccade.
